# Revealing Soil and Tree Leaves Deposited Particulate Matter PTE Relationship and Potential Sources in Urban Environment

**DOI:** 10.3390/ijerph181910412

**Published:** 2021-10-03

**Authors:** Gevorg Tepanosyan, Chiara Baldacchini, Lilit Sahakyan

**Affiliations:** 1 The Center for Ecological-Noosphere Studies of the National Academy of Sciences, Abovian-68, Yerevan 0025, Armenia; gevorg.tepanosyan@cens.am (G.T.); lilit.sahakyan@cens.am (L.S.); 2 Biophysics and Nanoscience Centre, Dipartimento di Scienze Ecologiche e Bioloigche (DEB)—Università degli Studi della Tuscia, Largo dell’Università snc, 01100 Viterbo, Italy; 3 Istituto di Ricerca Sugli Ecosistemi Terrestri-Consiglio Nazionale delle Ricerche (IRET-CNR), Via G. Marconi 2, 05010 Porano, Italy

**Keywords:** potentially toxic elements, urban trees, pollution, environmental compartments

## Abstract

Trees play a pivotal role in improving urban environmental quality and provide several ecosystem services including the removal of pollutants from the air, such as particular matter (PM) and potentially toxic elements (PTE). Therefore, understanding the tree PM and PTE capturing potential, also in connection with plant species, is of great concern, especially in urban areas. This study aims to reveal the link between the elemental composition of PM deposited on tree leaves and soils PTE contents, as well as to rank the PM capturing efficiency of 10 different tree species growing under the impact of urban environments. This also allowed us to test the efficiency of PM deposited on tree leaves as a PTE biomonitoring and pollution source identification tool, in the two biggest urban areas of Armenia. Indeed, high contents of PTE are detected in both soil- and leaf-deposited PM from sites characterized by the presence of localized and active pollution sources (i.e., industrial unites, high traffic, etc.), which are identified by specific tracers (such as Mo, Cu, Zn, Pb, and Cd). Among the studied tree species, the highest PM amount per unit leaf area is observed for *Platanus orientalis*, but elm species are also identified as promising canditates to be considered for their PM removing potential, and need to be included in future more details studies.

## 1. Introduction

The quality of the urban environment is a key factor affecting sustainable development and ensuring a better life for the urban population. According to the United Nations (UN), it is projected that 68% of the world’s population will live in urban areas by 2050 [1], and cities will become the nucleus of the transport, trade and information flows, where the highest quality of the public and private services are presented. However, the energy consumption and emission quantities are changing the chemical environment of cities, leading to the pollution of different environmental compartments [2], especially by potentially toxic elements (PTE). It is well known that the atmospheric environment is the “cradle” of all emissions, from which PTE, linked to airborne particulate matter (PM), migrate to other environmental compartments [3]. Moreover, it is already a fact that environmental pollution is a recognized menace in Europe, especially in the case of soils [4], which can serve as a secondary source of PTE contents’ introduction to the atmospheric environment through soil resuspension. Hence, it is crucial to study and address the changes occurring in the atmospheric chemical environment of urban areas, and to find optimal solutions for pollution prevention and risk mitigation conditioned by the PM-linked PTE contents. In recent years, several studies [5,6,7,8,9,10,11,12,13,14,15,16,17,18] tested different tree species and other urban greenery for their capacity in PM removal, and found that trees can serve as efficient nature-based solutions (NBS) for air quality management in urban areas. However, the leaf macro- and micro-structure plays a pivotal role in PM capture [10,13], resulting in dramatically different efficiencies among tree species. Muhammad et al., (2019) [19] found that among the 96 studied species, the most effective species for PM immobilization are *Buddleja davidii*, *Viburnum lantana*, and *Sorbus intermedia*, whereas the least effective were *Populus alba*, *Robinia pseudoacacia*, and *Abies fraseri*. In another study, the largest accumulation of PM_2.5_ was reported for coniferous species [18]. Dadea et al., (2017) [8] found that, from the perspective of heavy metal bioindication and phytoremediation, the most suitable among the nine studied species were fast-growing *Betula pendula* and *Robinia pseudoacacia*. However, it should be mentioned that several factors may affect the results presented in the literature, ranging from rainfall [13] to the analytical techniques used [20,21]. In addition, the studied tree species reported in the previous literature strongly depend on the geographical regions under investigation. Hence, it is very important to continue the study of other species, and expand the list of the urban greenery suitable for the bioindication and phytoremediation of air quality in urban environment. To fill this gap, this study investigates the spatial distribution patterns of PTE in PM deposited on tree leaves and in adjacent soils of Armenia’s two biggest cities, Yerevan and Gyumri, and tests the potential of tree species specific to Armenia’s urban environments for PTE biomonitoring and pollution source identification.

## 2. Materials and Methods

### 2.1. Study Sites

The territory of the capital city of Yerevan (latitude 40°10′40″ N, altitude 44°30′45″ E, 223 km^2^) covers an area represented by semi-desert, arid steppe and steppe landscapes, and is situated at 850–1420 m a.s.l. The climate is continental. In summer, the temperature ranges from +22 to +26 °C, and in winter it ranges from −20 to −30 °C. The annual mean precipitation level is 300–350 mm [22]. The city relief is dissected, and it is represented by plains, plateaus and foothills situated in the left and right sides of the river Hrazdan canyon. The geological basis of the city area is mainly represented by volcanic lavas, tuffs and quaternary sediments on which a mostly brown semi-desert soil profile is developed [23]. For more than half a century, Yerevan has been the most industrialized (approximately 60% of the country industry) city in Armenia. During the Soviet era, the city’s territory hosted several factories (i.e., an aluminum plant, an experimental plant for milling machines, and a typography and polygraphic complex, etc.), which led to the continuous release of PTE into the city environment and their accumulation in different environmental compartments [23,24,25,26]. However, the collapse of the Soviet Union led to a decrease in industrial production, and many factories were closed [23,27]. Today, Yerevan’s territory’s soil has retained the chemical signature of this historical pollution, and serves as a sink for the newly introduced PTE. Among these is Mo [28], whose presence in Yerevan’s atmosphere is related to the emissions from the “Plant of Pure Iron” [29].

The city of Gyumri (latitude 40°47′0″ N, altitude 43°50′0″ E, 44.41 km^2^) is situated in the central part of the Shiraki Plateau, at 1550 m a.s.l. [22]. The climate is continental. The warmest month of the year is August, with the mean temperature of +19 °C, and the coldest is January, with a mean temperature of −9 °C [30]. The annual mean precipitation level is 500 mm. The geological basis of the city is characterized by alluvial and proluvial sediments and by volcanic tuffs. The city’s territory is represented by chernozems [31]. During the Soviet era, more than 50 industrial units (bicycle plant, mechanical engineering, workshops of metal plating and paint and lacquer coating, textile production, etc.) were operating in Gyumri. However, in 1988, the city of Gyumri was destroyed by the Spitak Earthquake, and to this day the city’s territory carries the remnants of the disaster (collapsed buildings, debris and other garbage dumps, etc.) [32], which are a significant sources of dust and associated PTE.

### 2.2. Sampling

**Figure 1:**
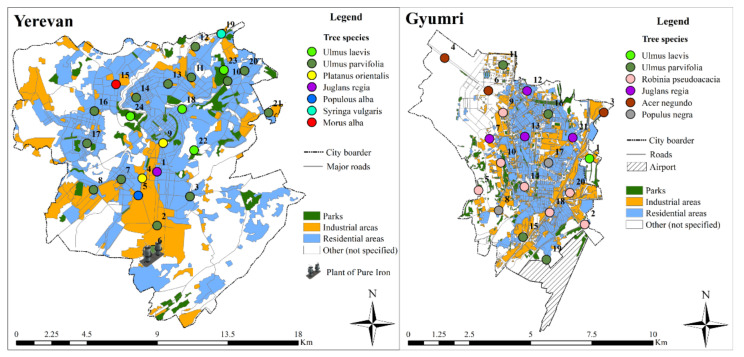
Sampling sites’ distribution in the Yerevan and Gyumri territories.

Adjacent samples of tree leaves and soils were collected in 24 and 21 sampling sites distributed in the main residential, industrial and green areas of the cities of Yerevan and Gyumri (Figure 1), respectively. The most widespread tree species were selected for the study: in Yerevan, leaves were sampled from *Ulmus laevis* (*Ul*, 4 sites), *Ulmus parvifolia* (*Up*, 14 sites), *Platanus orientalis* (*Po*, 2 sites), *Juglans regia* (*Jr*, 1 site), *Populous alba* (*Pa*, 1 site), *Syringa vulgaris* (*Sv*, 1 site) and *Morus alba (*Ma*, 1 site)* trees, while *Ulmus laevis* (*Ul*, 1 site), *Ulmus parvifolia* (*Up*, 4 sites), *Robinia pseudoacacia* (*Rp*, 7 sites), *Juglans regia* (*Jr*, 4 sites), *Acer negundo* (*An*, 3 sites) *and Populous nigra* (*Pn*, 2 sites) leaves were sampled in Gyumri (Figure 1). Sites were selected to allow sampling replicates: i.e., isolated trees were not included in the sampling design, and leaves were sampled from 3 to 7 trees of the same species in the same site, either from grouped or in-line trees. The sampling was performed once per site, in the summer of 2011 in Yerevan, and the summer of 2013 in Gyumri.

Tree leaves were sampled at a height of 1.5–2 m above the ground. After sampling, leaves were placed in paper bags and transported to the laboratory. After being dried at room temperature, leaves were washed with deionized water (MilliQ) and the washing solution was then passed through vacuum filtration using a weighted ash free filter (filter paper, retention limit 2–3 μm). Then, filters were dried and sent to the laboratory for the chemical analysis. The surface area of each washed leaf was measured using ImageJ software [33].

In the case of soil sampling, a stainless-steel shovel was used to collect 3–5 subsamples from the upper 5 cm of the soil layer present in the surroundings of the trees. The randomly selected subsamples were mixed into composite samples, which were stored in polyethylene bags and transported to the laboratory. In laboratory, soil samples were processed in accordance with ISO−11464 [34]. Specifically, samples were air-dried, homogenized, sieved (<2 mm), milled and stored until further analysis.

### 2.3. Analytic Methods

The filters and soil samples were dissolved in nitric acid (1:1, *wt*/*wt*), then the solution was evaporated and MilliQ water was added to the residual solution until a volume of 20 mL was obtained. The contents of Cd, As, Pb, Cr, Ni, Co, Zn, Cu, Ag, Hg, and Mo (filters and soil, Yerevan) and Pb, Cr, Ni, Zn, Cu, and Mo (filters, Gyumri) were determined by atomic absorption spectrometry (AAnalyst 800 AAS PE, USA). Contents of Pb, Cr, Ni, Zn, Cu, and Mo in Gyumri soil were determined using X-ray fluorescence spectrometry (Olympus Innov-X-5000 (USA)). All analyses were performed at the Central Analytical Laboratory of CENS accredited by ISO IEC 17025. In the present work, PTE contents below detection limits were given a value 1/2 of the detection limit [2,35].

### 2.4. Tree Leaves Dust Load Estimation

To calculate the tree leaves’ total PM load (TPML), the following formula was used:

TPML (mg/cm^2^·day) = m_dust_/S·t, (1)

where m_dust_ is the dust weight, S is the surface area of the washed leaves, and t is the number of days between the last rain event and the leaf sampling.

### 2.5. Data Analysis and Geochemical Mapping

The descriptive statistics of studied PTE contents in PM deposited on tree leaves and in soils of Yerevan and Gyumri are presented in Table 1. The normality of data was checked by the Shapiro–Wilk test. Based on normality test results, Pearson or Spearman correlation was applied to reveal the potential links between the elements in leaves and soils. Principal component analysis (PCA) was applied to study the relationship and to reveal possible groups of interrelated PTE, in order to identify their potential sources. The maps of spatial distribution of the studied elements were created using ArcGIS 10.6 software. The contents of the studied elements were classified based on their quantiles.

## 3. Results and Discussion

### 3.1. Data Analyses and PTE Contents

Before the chemical analysis, the dust deposited on the filters was subjected to the calculation of TPML. The results show that in Yerevan’s territory, TPML ranges from 0.11 to 0.91 mg/cm^2^, with the mean of 0.49 mg/cm^2^ daily, whereas in Gyumri, TPML ranges from 0.005 to 0.048 mg/cm^2^, with the mean of 0.02 mg/cm^2^ daily. It is evident that the mean value of TPML in Yerevan is 24.5 times greater than in Gyumri, and this can be explained by the fact that the level of industry and construction activities in Yerevan exceeds that in the Shirak region, including Gyumri, by more than 10 times [22], as well as by the significantly high level of traffic in the Yerevan area. The spatial distribution of TPML (Supplementary Materials, Figure S1) in Yerevan shows that comparatively high (4th quantile) values are mainly located on the borders between residential and industrial areas, characterized by a relatively high density of major roads. In the case of Gyumri, comparatively high values form a spatial cluster stretching from north to west. It should be mentioned that this part of the city is characterized by the presence of industrial areas and debris of the 1988 earthquake, as well as buildings construction activities in process. In addition, the winds (Supplementary Materials, Figure S1) the predominate in the city’s territory can play an essential role in dust resuspension, distribution and deposition processes.

The descriptive statistics of the studied PTE content in PM deposited on tree leaves and in adjacent soils of Yerevan and Gyumri are presented in Table 1, and the spatial distribution of the studied elements are illustrated in Supplementary Materials, Figures S2–S5. The detailed inspection of the studied element distribution maps enables us to reveal that in many cases, comparatively high contents of several elements are observed in the same sampling sites, which are mainly located in the industrial areas. This suggests the presence of a single multi-elemental source or single-element sources of pollution in close proximity. It is noteworthy that Mo, Cu, Zn, and Pb display comparatively high maximum values both in the PM and in the soils of Yerevan, and a similar regularity was observed for Cu, Zn, and Pb in Gyumri. Cu, Zn, and Pb are known to be typical indicators of anthropogenic activities, and comparatively high contents of these elements are generally observed in the residential and industrial parts of other cities [36,37,38,39]. The exception related to Mo’s appearance, together with Cu, Pb, and Zn; in Yerevan, this can be explained by its source in the city territory. As was previously identified [28], the high content of Mo in the Yerevan territory has an anthropogenic origin related to the activities of the “Plant of Pure Iron”, which is located in the southern part of the city (Figure 1).

The presence of anthropogenically introduced quantities of Mo, As, Ag, Hg, Cd (Yerevan’s tree leaf-deposited PM), Mo and Cu (Yerevan’s soil), and Cu and Zn (Gyumri’s tree leaves) is also confirmed by the >100% value of the coefficient of variation (CV) [2]. The results of the Shapiro–Wilk tests of normality show that the contents of the studied elements in Yerevan’s tree leaf-deposited PM display a normal distribution for Ni and Zn, and lognormal distribution for As, Cr, Pb and Cu. In Yerevan’s soils, Co, Cr, Pb and Zn follow a normal distribution, while Cu and Cd display a lognormal distribution. In the case of Gyumri, normal distribution is observed only for soil Pb, Ni, Cr, and Zn contents, whereas all the PTE contents of Gyumri’s tree leaf-deposited PM follow a lognormal distribution. In the cases of all other studied elements in both cities and both mediums, deviation from normality is detected.

**Figure 2:**
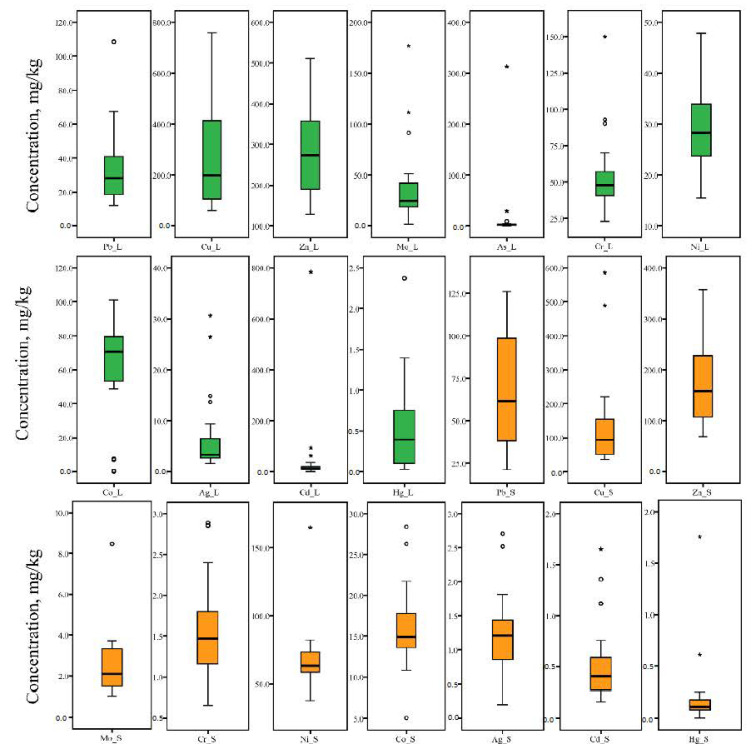
Box plots of studied PTE concentration in Yerevan’s tree leaf-deposited PM and soil (o—outliers and *—extreme values; for a better visualization, the extremely high content of Mo in sampling site 6 was removed).

**Figure 3:**
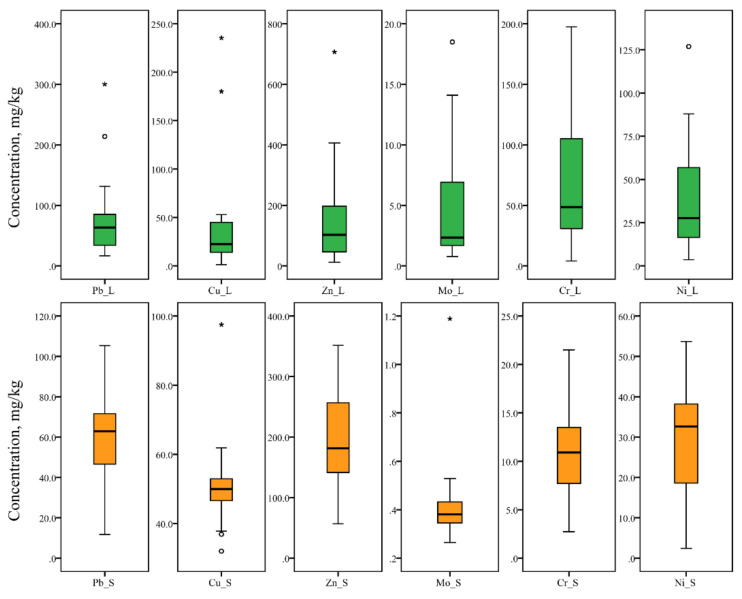
Box plots of studied PTE concentration in Gyumri’s tree leaf-deposited PM and soil (o—outliers and *—extreme values).

Box-plot analysis (Figure 2 and Figure 3) allows us to reveal the outliers and extreme values. The detailed inspection of outliers and extreme values highlights multi-elemental anomalies in the Yerevan city area. In particular, PM from the tree leaves of five sampling sites (namely, 2, 3, 5, 6 and 17 (Figure 1)) displays outliers and extreme values for three or more elements. Among these, sites 2, 3, 5, and 6 form a spatial cluster in the southern industrial part of the city. Moreover, site number 6 is located near the “Plant of Pure Iron”, which produces ferromolybdenum, molybdenum powder, metal molybdenum, etc. [29]. Thus, the “Plant of Pure Iron” is a potential source of Mo and Mo accessory elements involved in concentrate and its treatment process. An identical pattern was observed in the case of soil samples. In the case of Gyumri, a multi-elemental anomaly is identified in the sampling site number 14, where outliers and extreme values are displayed for Pb, Ni, and Cu. The site is located in the central historical part of the city, where, in addition to intensive traffic, several operating smitheries are also present.

### 3.2. Correlation Analysis of PTE Concentration

Considering the low number of samples and the presence of not normally distributed elemental concentrations in the datasets, Spearman’s correlational analysis was performed (Supplementary Materials, Tables S1 and S2).

In the case of Yerevan, Site 6 was excluded from the dataset, due to the unusually high contents of PTE associated with the Mo pollution source. Then, for tree leaf-deposited PM element contents (Supplementary Materials, Table S1), significant positive correlations were observed between Ni, Cr and As; As and Ag; Ag and Mo, Zn; Zn and Hg, Cd, Cu; Cu and Hg. In the case of soil (Supplementary Materials, Table S1), a significant positive correlation was detected between Cr and Ni, Mo; Pb and Ag, Zn; Zn and Cd, Cu; Cu and Cd. A significant negative correlation was observed between Pb and Ni. The correlation analysis between Yerevan’s leaf-deposited PM and soil PTE contents shows that a significant positive correlation exists between Cd leaf-deposited PM content and Cd, Zn, and Cu soil contents, between Zn leaf-deposited PM content and Zn, Cd, Pb, and Cu soil contents, and between Cu leaf-deposited PM content and Cu soil contents. Interestingly, the mutual correlation between leaf-deposited PM and soil Cd, Cu and Zn indicates that these elements have a similar origin in both mediums. The three elements are known to be produced due to car component corrosion and tire abrasion [40,41], thus suggesting that their presence on tree leaves is mainly due to dust resuspension. However, the Zn PM contents correlate significantly also with the soil contents of Zn and Pb, which are known to be historical pollutants of the Yerevan city environment [28]. Therefore, some quantities of Zn in PM can be also attributed to the resuspension of the historical pollution in city soil. On the other hand, the link between Pb soil and PM contents is missing, indicating that Pb content in leaf-deposited dust is mainly due to different sources. In general, it is reported that more than 90% of plant Pb uptake can be attributed to atmospheric sources [42]. Finally, the significant correlation between Ni and Cr indicated their possible geogenic origin in both mediums. However, the absence of the significant correlation between Ni and Cr’s respective contents in PM and soil suggests differences between the uptake mechanisms and sources of these elements in soils and tree leaf-deposited dust.

The relationship between the contents of Mo, Pb, Ni, Cr, Zn, and Cu in Gyumri’s tree-leaf deposited PM and adjacent soil (Supplementary Materials, Table S2) differs from the patterns identified in Yerevan. Particularly, in tree leaves, a significant positive mutual correlation is detected between Mo, Pb, Ni, Cr, and Zn and Cu and Pb. In soils, significant a positive mutual correlation is observed between Mo, Pb, Cr, and Zn. Mo also correlates with Ni, while Pb, Cr, and Zn correlate with Cu. Ni significantly correlates with Cr and Zn. The similar pattern of mutual correlation of the studied elements observed in both mediums indicates their potential origination from the similar sources. Moreover, the unusual combination of mutually correlated elements and the detailed study of Gyumri’s soils [32] indicated their potential anthropogenic origin in the city’s territory. However, the elemental contents in tree leaf-deposited PM do not correlate with those in the soil. This could be the result of various features of elemental migration, accumulation and interaction with plants.

It is worth further noting that, in both cities and mediums, the link between Mo and Cu is missing, and this can be explained by their antagonistic behavior, especially in the case of plants [43].

### 3.3. Total PM Load (TPML) and Plant Species

**Figure 4:**
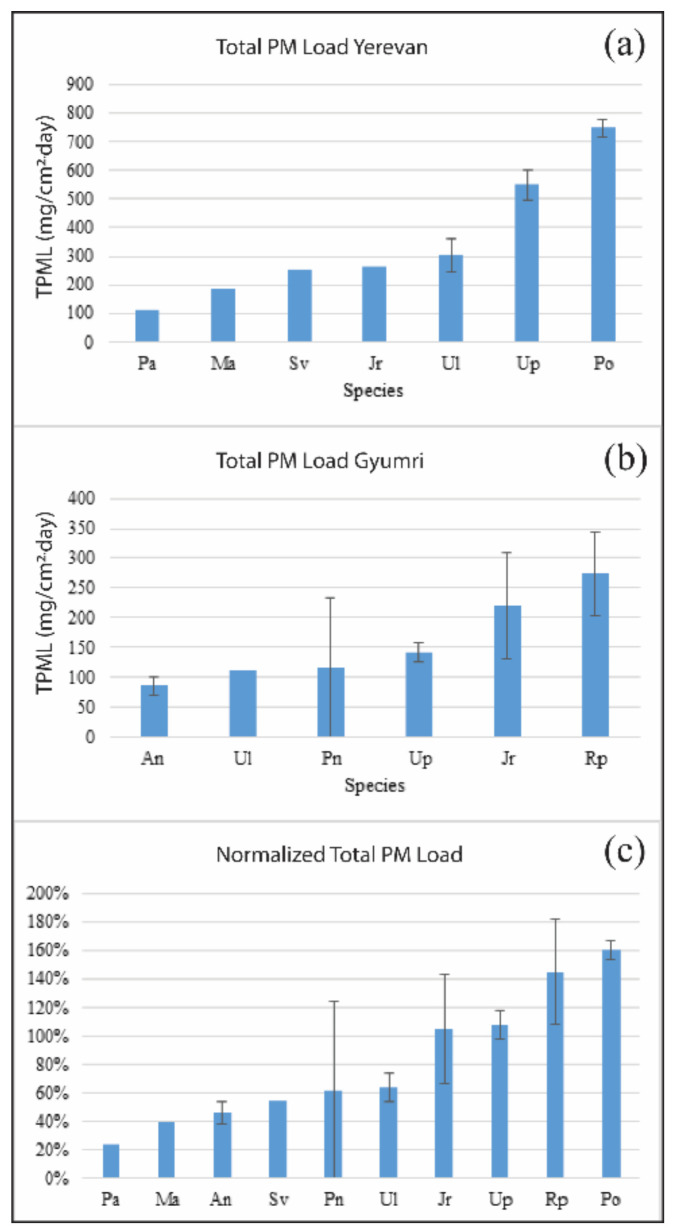
Average total PM load (TPML) per tree species in Yerevan (**a**) and Gyumri (**b**). The data from the two cities, properly normalized by the city average TPML, are averaged and combined in panel (**c**). Error bars represent standard deviations.

The relationship between tree species and TPML has been also explored. Indeed, it is known that the micro and macro morphological traits of tree leaves have an impact on the tree’s capacity for retaining PM particles on the surface of its leaves [7,10]. In Figure 4a,b, the average TPML per tree species (with standard deviation, when allowed by the site replicate number) is plotted, for Yerevan and Gyumri data, respectively. A clear trend is observed in both cases, with the less efficient species being able to remove about 1/7 and 1/3 of the amount demonstrated by most efficient species, in Yerevan and Gyumri, respectively. Three species are common among the two cities: *Ulmus leavis*, *Ulmus parvifolia,* and *Juglans regia*. *Ulmus leavis* has an average TPML lower than that of *Ulmus parvifolia* in both cases, while *Juglans regia* has an average TPML similar to *Ulmus leavis* in Yerevan (but this is a single site result) and similar to *Ulmus parvifolia* in Gyumri.

To compare all the sampled species in a single plot, each TPML value was normalized by the average TPML measured in the corresponding city, obtaining the normalized (or percentage) TPML. Then, all the normalized TPML values of the same tree species were averaged again, disregarding the city of sampling. The average normalized TPML values for each tree species are plotted, with their standard deviations, in Figure 4c. Many works ranking tree species based on their capacity for PM retention have been published previously [7,9,10,15,16,17,18]. However, only a few of the species studied in the present paper have been investigated previously. Thus, the present work represents an important contribution in the field, because it presents data on selected tree species for the first time (to the best of our knowledge). However, some features already observed are confirmed by our study; this further strengthens our approach.

For instance, *Platanus orientalis* has already been presented as a highly efficient species in PM capturing [17], and *Juglans regia* and *Syringa vulgaris* have both already been reported as being moderately efficient, with similar PM capturing capability, with a slight predominance of the former [9]. Moreover, among the 96 studied species in [9], *Ulmus glabra* was found to be the ninth most effective species for PM immobilization; this confirms that elm species are good PM capturers, as we observed in our study for *Ulmus laevis* and *Ulmus parvifolia* (Figure 4c). Among the selected species, *Robinia pseudoacacia* is probably the most studied one. Experimentally, it has been observed that this species is less efficient in PM capturing [9,10]. In particular, it has been reported to capture less PM than *Juglans regia* and *Syringa vulgaris* [9], and almost the same amount of *Populus nigra* [10]. Both pf these results are in contrast with our observations, but are in line with the results provided in Dadea et al., (2017). However, these results were obtained with different techniques (namely, remanent magnetization [9] and electron microscopy [10]). Moreover, a high PM capturing capability from *Robinia pseudoacacia* is conversely in agreement with the complexity of the micro and macro morphological traits of its leaves [10].

### 3.4. Principal Component Analysis (PCA)

PCA was run, for both cities separately, by using as the input variable the elemental contents of both leaf-deposited dust and soil.

**Figure 5:**
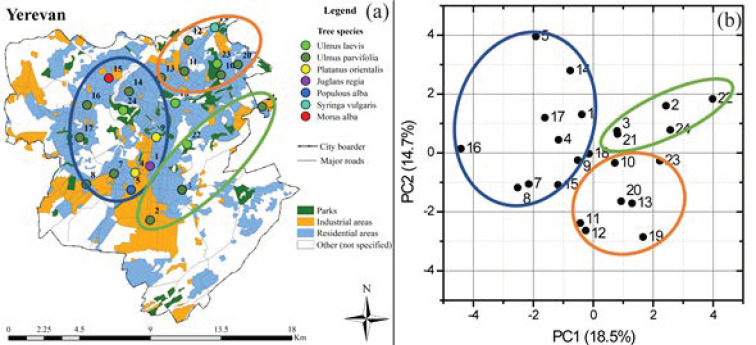
Yerevan territory tree leaves and soil element contents PCA results-spatial representation (**a**) and factor scores per case (**b**).

In the case of Yerevan, PCA was run for all the selected sites, except for site 6 (because it is an outlier in Mo contents). Besides As, which was only detected in leaves, the contents of Ni, Co, Ag, Hg, Cr, Pb, Mo, Cd, Zn, and Cu were used as input variables, and 20 principal components (PCs) were obtained. The first two PCs explain 18.5% and 14.7% of the total variance, respectively. The PC1 and PC2 factor-variable correlation coefficients are reported in the Supplementary Materials, Table S3. Briefly, PC1 separated the sites with high contents of Ni, Cr, Co and Mo both in leaves and in the soil and the sites with high Hg contents in the soil (positive PC1 values) from the others. PC2 further separated these sites, having those with high Hg contents in its negative region and those with high Mo contents in the positive region. As a result, based on the factor scores per case, a good geographical separation of the sites was identified (Figure 5). In particular, the Hg soil contents separate the sites in the north-east corner (orange group) from the others. Indeed, the north-east part of the city is the area where comparatively high soil Hg contents are observed (Supplementary Materials, Figure S3).

The sites with high Mo contents both in the soil and on the leaves are grouped in the green cluster, which is located close to the “Pure Iron” plant in the southern industrial areas of Yerevan (Figure 5 and Supplementary Materials, Figure S3), with the exception of site 24. The remaining sites are grouped into the blue cluster, which is characterized by the dominance of the western residential areas of the city, where comparatively high contents of Zn and Ag on tree leaves and Pb, Zn, Ag in soil are observed. Noticeably, site 18 is in between the blue and the orange group, both in the map and in the PCA plot. This site is located in a residential area that, according to data collected during the sampling campaign, is characterized by a high level of traffic and displays comparatively high values of Hg and Cr in leaves and Hg and Zn in soils.

In the case of Gyumri, PCA was run for all the sites, and by using the Ni, Cr, Pb, Mo, Zn, and Cu contents in both leaves and soil, 12 PCs were thus obtained, with the first two representing 37.3% and 28.2% of the total variance, respectively. The factor-variable correlation coefficients are reported in the Supplementary Materials, Table S3. However, the projection of cases in the PCA factor scores PC1–PC2 plane (not shown) only separates sites with highly polluted leaves (positive PC1) from those with high soil contamination (negative PC2). Indeed, as also shown by the correlation analysis, leaf and soil data, in contrast to Yerevan, do not correlate in the Gyumri dataset, and this can be explained by the possible superposition of the modern composition of emissions and the specificities of pollution sources operated before the 1988 earthquake and the remaining debris distributed in the city’s territory in the following decades, which are specifically reflected in the soil, whereas leaves reflect the modern situation in the city territory.

## 4. Conclusions

The study of the PTE contents in PM deposited on tree leaves and nearby soil, in Yerevan and Gyumri, showed that in both cities and both mediums, comparatively high maximum values are observed for Cu, Pb, and Zn. A significant positive relationship between tree leaf-deposited PM and soil contents of Cu, Zn, and Cd has been obtained in Yerevan. In the case of Gyumri, although the link between tree leaves and soil was missing, a similar intercorrelation between the studied PTE contents is observed in each medium. In addition, in Yerevan, conditioned by the activities of the “Plant of Pure Iron”, Mo also displays similar regularity in soil and airborne PM. Moreover, it was revealed that the spatial pattern of the studied PTE and detected outliers and extreme values are in line with the industrial areas and residential areas with high level of traffic and known sources of pollution. Indeed, through PCA, the data from Yerevan revealed obvious spatial separation of the study sites and enabled us to identify potential sources of pollution. Finally, the assessment of TPML on leaves allowed us to rank the selected species according to their PM capturing ability, which decreases in the following order: *Platanus orientalis* > *Robinia pseudoacacia* > *Ulmus parvifolia* > *Juglans regia* > *Ulmus laevis* > *Populous nigra* > *Syringa vulgaris* > *Acer negundo* > *Morus alba* > *Populous alba*. The results of this study thus deserve interest as a basis for city planners to use urban greening as an effective means for bioindication, pollution prevention and mitigation toobtain a better urban environmental quality, especially in the Caucasian region, due to the tree species selection performed.

## Figures and Tables

**Table 1:** The descriptive statistical parameters of the studied chemical elements (mg/kg) and the coefficient of variation (CV, %) in Yerevan and Gyumri.

Elements	Mean	St. Er. of Mean	Median	Min.	Max.	Skew.	CV, %
Yerevan leaves							
Ni_L	28.9	1.53	28.4	15.5	47.8	0.4	26
Co_L	61.5	6.06	70.6	0.1	101	−1.1	48.3
As_L	16.3	13	1.98	0.035	313	4.8	390
Ag_L	6.56	1.56	3.34	1.56	30.6	2.3	116
Hg_L	0.546	0.119	0.394	0.03	2.37	1.6	106
Cr_L	54.2	5.35	48	23.1	150	2.4	48.3
Pb_L	32.3	4.31	28.3	12.1	108	2.3	65.3
Mo_L	1057	1017	27.4	1.54	24,456	4.9	472
Cd_L	50	32.2	12.1	0.088	783	4.8	315
Zn_L	280	23.5	274	129	511	0.6	41.1
Cu_L	288	47.6	198	60.2	758	0.9	81
Yerevan soil							
Ni_S	68.2	4.66	63.2	38.2	164.7	3.4	33.5
Co_S	16	1.01	14.9	5.07	28.4	0.6	30.9
Ag_S	1.23	0.116	1.21	0.202	2.71	1	46.1
Hg_S	0.26	0.11	0.12	0.064	1.76	3.43	164
Cr_S	1.56	0.116	1.47	0.661	2.89	0.9	36.4
Pb_S	67.5	6.96	61.6	21.1	126	0.3	50.5
Mo_S	169	166	2.2	1.04	3986	4.9	483
Cd_S	0.512	0.077	0.407	0.16	1.65	1.8	74
Zn_S	175	16.8	158	68.8	357	0.7	47
Cu_S	132	27.8	94.1	36	586	2.5	103
Gyumri leaves							
Mo_L	4.88	1.06	2.34	0.78	18.5	1.6	99.5
Pb_L	77.3	15.1	63.5	16.6	300	2.1	89.8
Ni_L	40.8	6.76	27.6	3.6	127	1.3	76
Cr_L	68.3	11.5	48.6	4.05	198	1.1	76.9
Cu_L	43.7	12.5	22.4	1.25	235	2.7	131
Zn_L	158	35.4	103	11.9	707	2.2	102
Gyumri soils							
Mo_S	0.42	0.041	0.381	0.265	1.19	3.8	44.4
Pb_S	59.4	5.64	62.9	11.7	105	−0.1	43.5
Ni_S	28.3	2.93	32.6	2.4	53.7	−0.2	47.4
Cr_S	10.7	1.07	10.9	2.72	21.5	0.3	45.6
Cu_S	50.8	2.82	50	32	97.5	2.3	25.5
Zn_S	185	17.2	182	56.6	352	0.2	42.5

## Data Availability

Data sharing not applicable.

## Supplementary Materials

The following are available online at https://www.mdpi.com/article/10.3390/ijerph181910412/s1, Figure S1: Tree leaves particulate matter (PM) loads distribution and wind roses for the cities of Yerevan and Gyumri, Figure S2: Spatial distribution in Yerevan of the content of potentially toxic element (PTE) in tree leaves, Figure S3: Spatial distribution in Yerevan of the content of potentially toxic element (PTE) in soil, Figure S4: Spatial distribution in Gyumri of the content of potentially toxic element (PTE) in tree leaves, Figure S5: Spatial distribution in Gyumri of the content of potentially toxic element (PTE) in soil, Table S1: Spearman correlation matrix of element concentration data in Yerevan soil, Table S2: Spearman correlation matrix of element concentration data in Gyumri soil, Table S3: Factor-variable correlation coefficients for the first two principal components (PC) obtained by a principal component analysis (PCA) with the element concentration data in soil and leaves as input variables, for both Yerevan and Gyumri.

## Availability Data and Materials

The datasets used and/or analyzed during the current study are available from the corresponding author on reasonable request.
