# Supplementary material for: Revealing Soil and Tree Leaves Deposited Particulate Matter PTE Relationship and Potential Sources in Urban Environment
Source: Int J Environ Res Public Health. 2021 Oct 3;18(19):10412. doi: 10.3390/ijerph181910412 (PMC8508361; doi:10.3390/ijerph181910412)
Supplement: Supplementary file 1 [file ijerph-18-10412-s001.zip › ijerph-1391894-supplementary.pdf]

Revealing soil and tree leaves deposited particulate matter PTE relationship and potential sources in urban environment

Gevorg Tepanosyan[1], Chiara Baldacchini[2,3,*] and Lilit Sahakyan[1]

1 The Center for Ecological-Noosphere Studies of the National Academy of Sciences, Yerevan 0025, Abo-vian-68, Republic of Armenia
2 Biophysics and Nanoscience Centre Dipartimento di Scienze Ecologiche e Bioloigche (DEB) - Università degli Studi della Tuscia, Largo dell'Università snc, 01100 Viterbo, Italy
3 Istituto di Ricerca sugli Ecosistemi Terrestri - Consiglio Nazionale delle Ricerche (IRET-CNR), Via G. Marconi 2, 05010, Porano, Italy
*Corresponding author: baldacchini@unitus.it

SUPPLEMENTARY MATERIALS

- **SM Figure S1** Tree leaves particulate matter (PM) loads distribution and wind roses for the cities of Yerevan and Gyumri.

- **SM Figure S2** Spatial distribution in Yerevan of the content of potentially toxic element (PTE) in tree leaves.

- **SM Figure S3** Spatial distribution in Yerevan of the content of potentially toxic element (PTE) in soil.

- **SM Figure S4** Spatial distribution in Gyumri of the content of potentially toxic element (PTE) in tree leaves.

- **SM Figure S5** Spatial distribution in Gyumri of the content of potentially toxic element (PTE) in soil.

- **SM Table S1.** Spearman correlation matrix of element concentration data in Yerevan soil.

- **SM Table S2.** Spearman correlation matrix of element concentration data in Gyumri soil

- **SM Table S3.** Factor-variable correlation coefficients for the first two principal components (PC) obtained by a principal component analysis (PCA) with the element concentration data in soil and leaves as input variables, for both Yerevan and Gyumri.

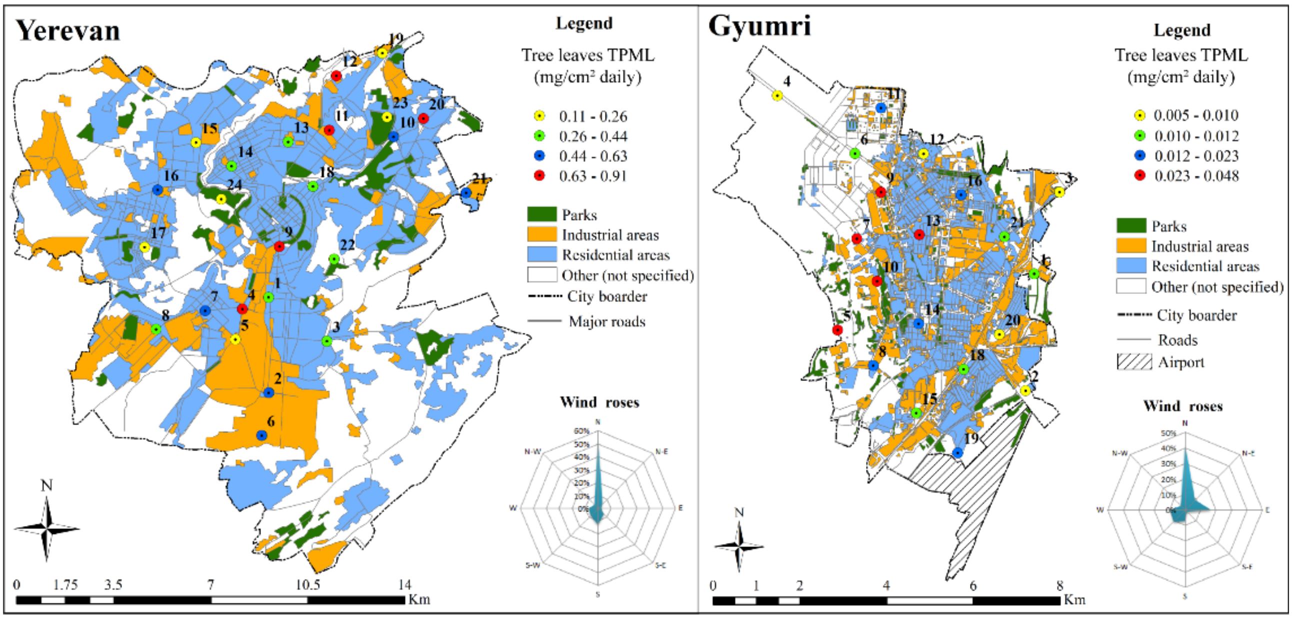

SM Figure S1. Tree leaves particulate matter (PM) loads distribution and wind roses for the cities of Yerevan and Gyumri.

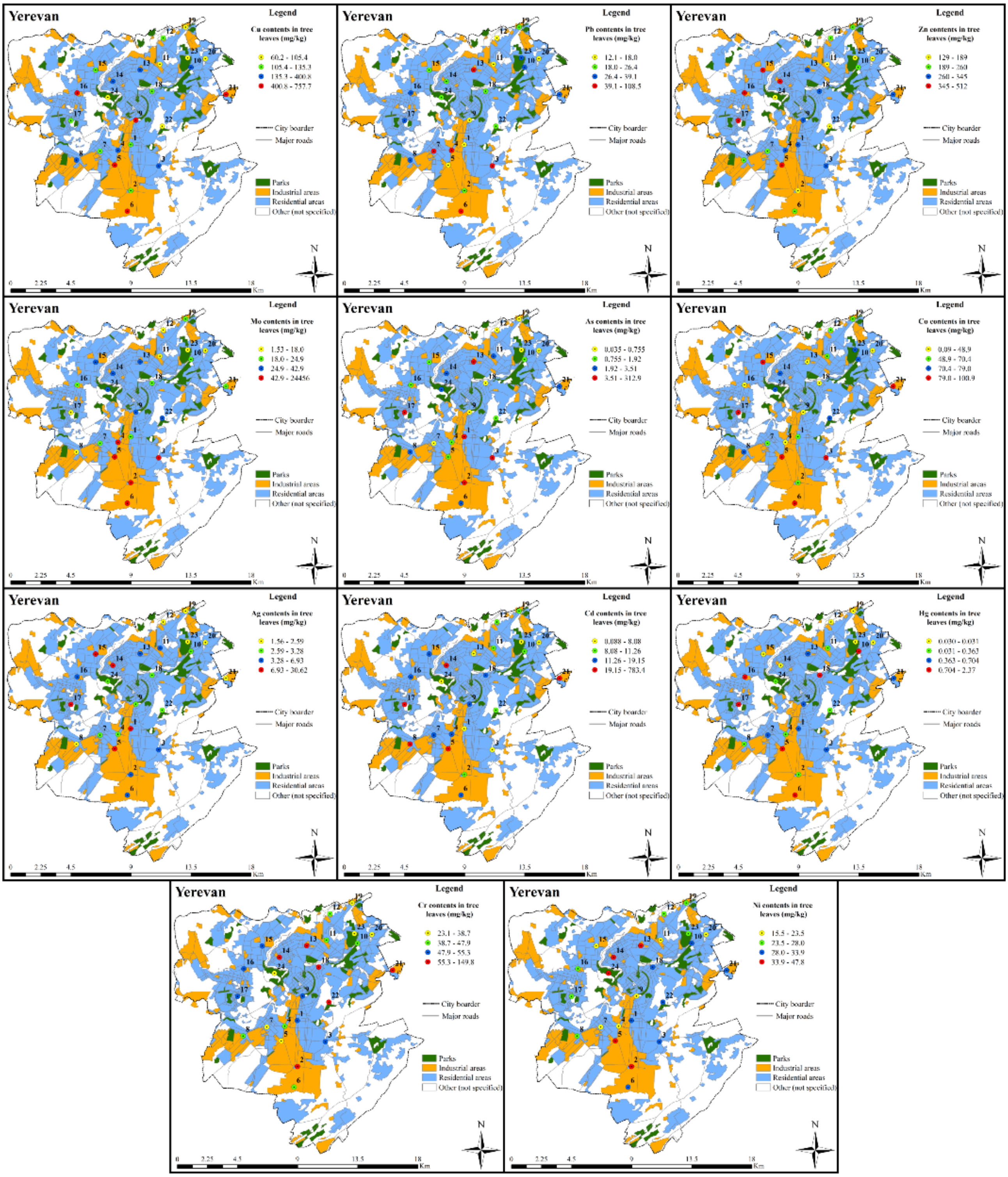

SM Figure S2. Spatial distribution in Yerevan of the content of potentially toxic element (PTE) in tree leaves.

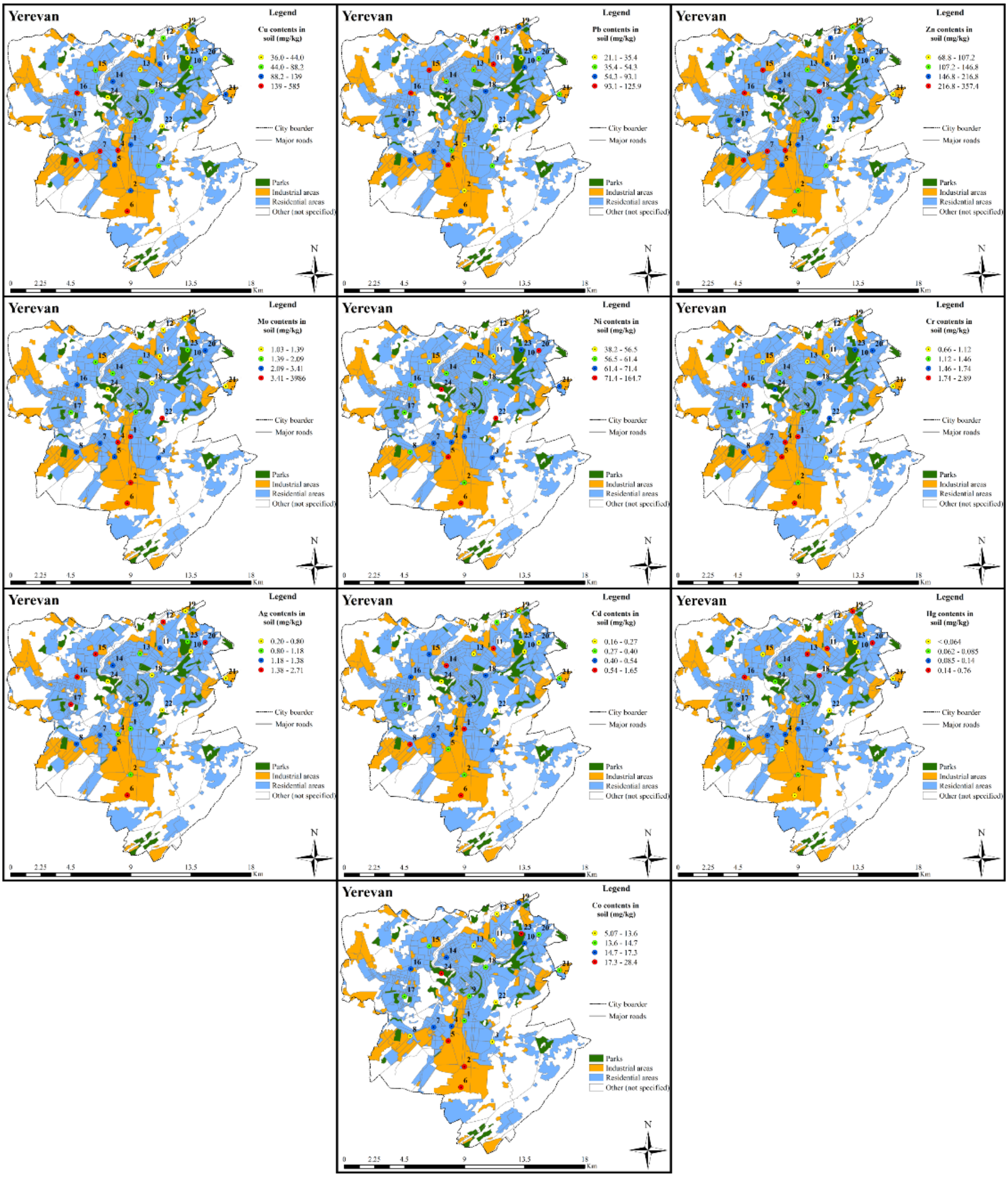

SM Figure S3. Spatial distribution in Yerevan of the content of potentially toxic element (PTE) in soil.

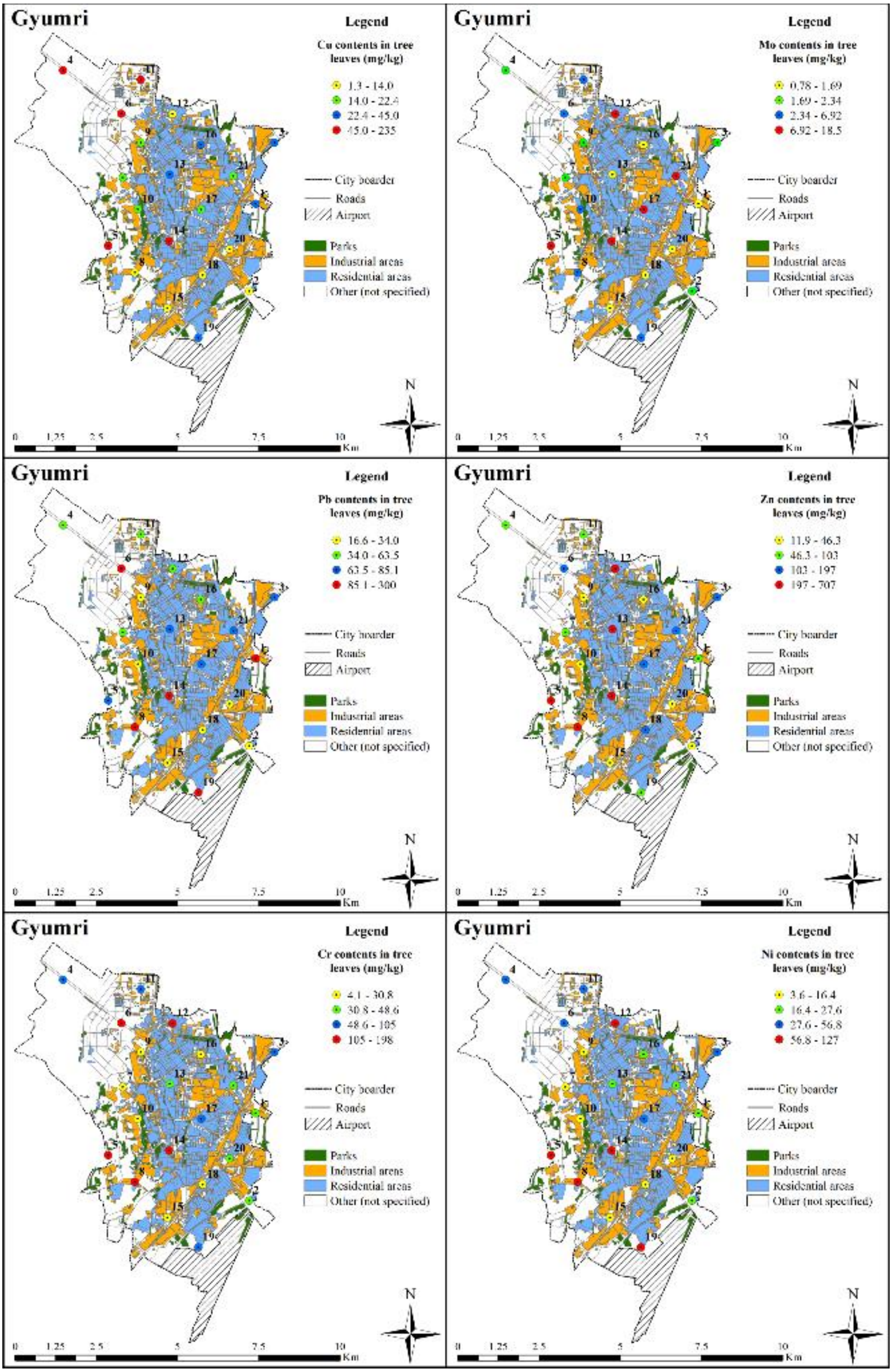

SM Figure S4. Spatial distribution in Gyumri of the content of potentially toxic element (PTE) in tree leaves.

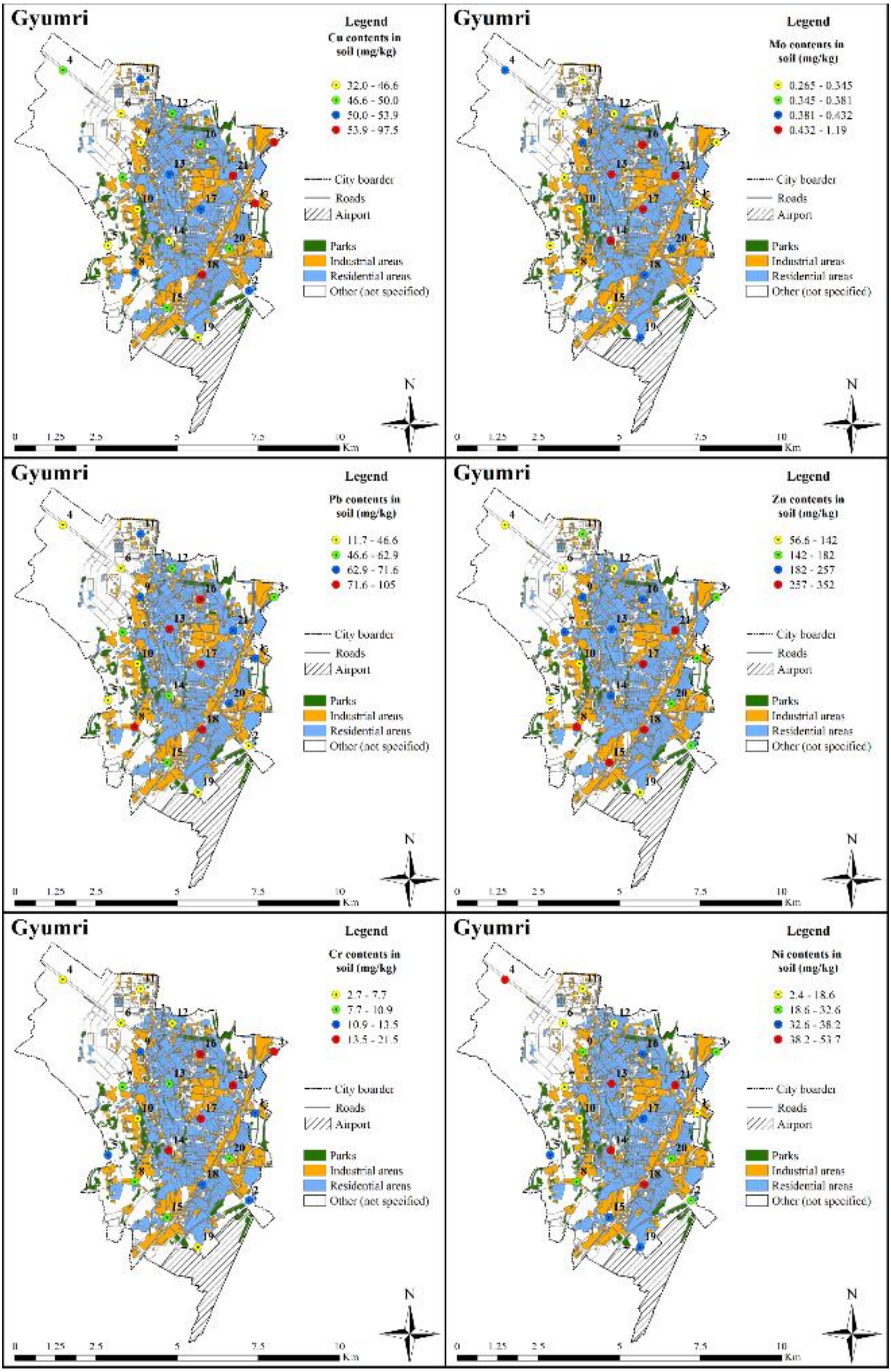

SM Figure S5. Spatial distribution in Gyumri of the content of potentially toxic element (PTE) in soil.

SM Table S1. Spearman correlation matrix of element concentration data in Yerevan soil.

Parameters	Ni_L	Co_L	As_L	Ag_L	Hg_L	Cr_L	Pb_L	Mo_L	Cd_L	Zn_L	Cu_L	Ni_S	Co_S	Ag_S	Cr_S	Pb_S	Mo_S	Cd_S	Zn_S	Cu_S
**TDL_L**																				
**Ni_L**	1.000																			
**Co_L**	.330	1.000																		
**As_L**	**.585****	.290	1.000																	
**Ag_L**	.363	.185	**.574****	1.000																
**Hg_L**	.396	-.050	.268	.311	1.000															
**Cr_L**	**.448***	-.026	**.437***	.261	.196	1.000														
**Pb_L**	.003	.188	.320	-.048	.127	-.048	1.000													
**Mo_L**	.376	.193	.292	**.511***	.082	.293	.090	1.000												
**Cd_L**	-.022	.159	.011	.203	.287	.281	.042	.019	1.000											
**Zn_L**	.128	.115	.226	**.596****	**.482***	.290	.102	.206	**.659****	1.000										
**Cu_L**	.170	.061	.274	.326	**.554****	.219	.254	.311	**.593****	**.694****	1.000									
**Ni_S**	.149	.303	.028	-.013	.066	-.217	-.041	.187	-.041	-.106	.000	1.000								
**Co_S**	.190	.205	-.044	.194	.052	-.283	.035	.305	.077	-.042	.123	.351	1.000							
**Ag_S**	-.339	.016	-.079	.205	-.192	-.143	-.047	-.184	.234	.328	.171	-.056	-.226	1.000						
**Cr_S**	-.021	-.215	-.168	-.026	.074	-.229	-.128	-.010	.063	.005	.064	**.689****	.402	-.124	1.000					
**Pb_S**	-.254	.010	-.217	.026	.182	-.165	.150	-.172	.408	**.423***	.254	**-.466***	-.271	**.454***	-.373	1.000				
**Mo_S**	-.073	-.059	.133	.189	-.200	.187	-.002	.273	-.015	.022	.034	.397	-.063	.276	**.469***	-.284	1.000			
**Cd_S**	-.200	-.147	.193	.313	-.004	.173	.024	-.020	**.441***	**.477***	.344	-.164	-.152	.319	.099	.294	.270	1.000		
**Zn_S**	-.255	-.263	-.044	.178	.049	.058	.223	-.081	**.535****	**.599****	.361	-.297	-.158	.374	.129	**.608****	.208	**.771****	1.000	
**Cu_S**	-.132	-.051	.176	.221	.207	.060	.209	.114	**.531****	**.419***	**.578****	.073	.080	.319	.217	.392	.347	**.756****	**.665****	1.000
**Hg_S**	-.116	**-.609****	-.009	.163	.051	-.050	-.015	-.048	-.208	.085	-.236	-.304	-.155	-.057	-.062	.022	.097	.054	.211	-.104

**. Correlation is significant at the 0.01 level (2-tailed).

*. Correlation is significant at the 0.05 level (2-tailed).

SM Table S2. Spearman correlation matrix of element concentration data in Gyumri soil.

Parameters	Mo_L	Pb_L	Ni_L	Cr_L	Cu_L	Zn_L	Mo_S	Pb_S	Ni_S	Cr_S	Cu_S
**TDL_L**											
**Mo_L**	1.000										
**Pb_L**	**.526***	1.000									
**Ni_L**	**.762****	**.847****	1.000								
**Cr_L**	**.808****	**.801****	**.961****	1.000							
**Cu_L**	.192	**.494***	.401	.330	1.000						
**Zn_L**	**.605****	**.714****	**.794****	**.820****	.290	1.000					
**Mo_S**	-.112	.073	-.057	-.062	.227	.060	1.000				
**Pb_S**	-.395	-.108	-.274	-.310	-.263	-.016	**.482***	1.000			
**Ni_S**	-.070	-.008	.049	.036	.077	.281	**.585****	.296	1.000		
**Cr_S**	-.003	.137	-.016	-.027	.075	.141	**.459***	**.479***	**.462***	1.000	
**Cu_S**	-.276	.010	-.207	-.138	-.288	.015	.181	**.542***	.154	**.466***	1.000
**Zn_S**	-.271	-.153	-.268	-.260	-.292	.055	**.449***	**.786****	**.438***	**.541***	**.563****

**. Correlation is significant at the 0.01 level (2-tailed).

*. Correlation is significant at the 0.05 level (2-tailed).

SM Table S3. Factor-variable correlation coefficients for the first two principal components (PC) obtained by a principal component analysis (PCA) with the element concentration data in soil and leaves as input variables, for both Yerevan and Gyumri.

Variables	PC1	PC2
	**Yerevan**	
Ni_levaes	0,323	0,458
Co_ levaes	0,305	0,232
Ag_ levaes	-0,264	0,574
Hg_ levaes	-0,313	0,484
Cr_ levaes	0,405	0,357
Pb_ levaes	-0,095	0,017
Mo_ levaes	0,245	0,459
Cd_ levaes	-0,144	0,381
Zn_ levaes	-0,692	0,449
Cu_ levaes	-0,508	0,433
Ni_soils	0,118	0,600
Co_ soils	0,219	0,451
Ag_ soils	-0,428	-0,145
Hg_ soils	0,182	-0,455
Cr_ soils	-0,191	0,444
Pb_ soils	-0,693	-0,309
Mo_ soils	0,226	0,382
Cd_ soils	-0,515	0,005
Zn_ soils	-0,836	-0,078
Cu_ soils	-0,723	0,019
	**Gyumri**	
Ni_leaves	0,969	0,188
Cr_leaves	0,953	0,165
Pb_leaves	0,781	0,008
Mo_leaves	0,662	0,071
Zn_leaves	0,939	-0,093
Cu_leaves	0,722	0,124
Ni_soil	0,286	-0,589
Cr_soil	0,275	-0,779
Pb_soil	-0,019	-0,680
Mo_soil	0,053	-0,754
Zn_soil	0,086	-0,785
Cu_soil	-0,099	-0,829
